# Rigor and reproducibility: status and challenges for single vesicle analysis

**DOI:** 10.20517/evcna.2022.28

**Published:** 2022-08-23

**Authors:** John P. Nolan, Daniel T. Chiu, Joshua A. Welsh

**Affiliations:** 1 Scintillon Institute, San Diego, CA 92121, USA.; 2 Cellarcus Biosciences, La Jolla, CA 92037, USA.; 3 University of Washington, Seattle, WA 98195, USA.; 4 Translational Nanobiology Section, Laboratory of Pathology, Center for Cancer Research, National Cancer Institute, Bethesda, MD 20892, USA.

**Keywords:** Extracellular vesicle, exosomes, flow cytometry, calibration, standardization, reproducibility

## Abstract

This report summarises the presentations and activities of the SELECTBIO Workshop on Rigor and Reproducibility in EV Research and Single EV Analysis held in San Diego, USA, in December 2021. The motivation for the session was the recognition that progress in the extracellular vesicle (EV) field is limited by the availability of rigorous and reproducible EV measurement tools. These tools are absolutely required for EVs to evolve from a research lab curiosity to something that will improve our ability to understand, diagnose, treat, and prevent disease. The program focused on guidelines for EV measurement and characterization as laid out in the recent MISEV2018 and MIFlowCyt-EV publications, their implementation in routine practice, and their continued evolution as new EV measurement technologies are introduced. The conclusion of the workshop was that more effort focused on pre-analytical issues and benchmarking of isolation methods is needed to strengthen collaborations and advance more effective biomarkers.

The recent SELECTBIO Conference in San Diego (December 12–14, 2021) included a Workshop on Rigor and Reproducibility in EV Research and Single EV Analysis as part of the Extracellular Vesicles: Technologies and Investigations track. The motivation for the session was the recognition that progress in the extracellular vesicle (EV) field is limited by the availability of rigorous and reproducible EV measurement tools. These tools are absolutely required for EVs to evolve from a research lab curiosity to something that will improve our ability to understand, diagnose, treat, and prevent disease. The program focused on guidelines for EV measurement and characterization as laid out in the recent MISEV2018^[1]^ and MIFlowCyt-EV^[2]^ publications, their implementation in routine practice, and their continued evolution as new EV measurement technologies are introduced.

The starting point for discussion was a review of the MISEV2018 reporting guidelines and the MIFlowCyt-EV reporting framework for single EV flow cytometry. Both of these reporting guidelines are intended as recommendations to increase rigor and reproducibility in the EV field, and also act as a tool for editors and reviewers to assess the strengths and weaknesses of manuscripts, proposals, funding applications, and conference presentations. The MISEV guidelines cover, at a high level, all aspects of EV research, from biofluid collection through EV and EV cargo characterization to EV functional analysis^[1]^. MIFlowCyt-EV addresses the reporting of essential details of single-EV flow cytometry analysis required for data interpretation and reproducibility, and is an extension of the general MIFlowCyt reporting framework^[3,4]^. Key elements of these reporting guidelines include: (1) the use of a calibrated instrument so that measurements can be reported in standardized and biologically meaningful rather than arbitrary units; (2) inclusions of several essential controls to demonstrate the specificity of EV detection and cargo measurement; and (3) comprehensive reporting of methods and results, including data sharing, to enable others to reproduce and extend the work. Workshop attendees were introduced to the significant amount of educational material on these topics available on the website of the EV Flow Cytometry Working Group (https://www.evflowcytometry.org/), a group of EV researchers affiliated with ISEV, ISAC, and ISTH that developed and promoted these guidelines^[5,6]^.

The discussion also covered challenges and opportunities for future advances in single-EV analysis. Rigorous and reproducible measurements of EV number, size and cargo by flow cytometry are possible^[2,7–9]^ and routine with commercial assay kits^[10–14]^, but instrument performance generally limits assay sensitivity. Most commercial flow cytometers have been designed to measure lymphocytes, but lab-built flow cytometers with single-molecule sensitivity have been possible for decades^[15–17]^, and may soon be commercially available. The general lessons for assay development and validation using these single-molecule sensitive instruments still apply, including the need to fully report methodological details, demonstrate the specificity of EV and cargo detection through the appropriate controls, and report data in the appropriate calibrated units. This last point generated additional discussion centered around how to reconcile “top-down” calibration approaches developed for the analysis of cells with the “bottom-up” approach enabled by single-molecule detection.

Flow cytometer fluorescence calibration was initially developed for cell analysis several decades ago, with standards, protocols and software to allow users to readily report particle brightness in standard units (MESF, mean equivalent soluble fluorochromes; ERF, equivalent reference fluorochromes; or ABC, antibodies bound per cell)^[18–21]^. Approaches for fluorescence calibration are robust for the measurement of bright particles such as cells, and have been shown to have utility in standardization when extrapolated to the measurement of dim particles such as EVs on suitably sensitive instruments. Along with fluorescence calibration, the development of light scatter calibration methods has shown utility for the standardization and characterization of small particles^[7,8,17,22,23]^. When detecting light from very small numbers of photons (e.g., ~100 fluorochrome molecules), additional factors arise and can affect the uncertainty of these calibrations^[7]^. One of these is the natural effect of counting small numbers of anything (including photons, molecules, or particles), where Poisson-type counting statistics introduce a known but stochastic uncertainty into the results. Another has to do with the ways different instruments handle background. Sensitive detection of any analyte is most often limited by background, and definitions of the lower limits of detection (LoD) often reference the variation in the background signal (e.g., LOD is the mean of the background +3 standard deviations), whether molecules or particles are being detected. Most commercial instruments “subtract” the constant background measured by the instrument, but they often perform this with different methods, and cannot remove the variation (or “noise”) in the background, which generally remains. Moreover, beads used for calibration often have their own intrinsic background autofluorescence, the measurement of which will depend on the sensitivity of the instrument. Thus, while the “top-down” calibration approaches developed for cell analysis can be extended into the realm of very dim EVs and the instrument limits of detection, care is required to understand the uncertainties and avoid over-interpretation.

By contrast, single-molecule detection starts with the detection of a single molecule, and a particle that is brighter than a single molecule should be able to be reported as having some number of single molecules (the “bottom-up” approach)^[24]^. In practice, for single molecule-based detection approaches, issues of Poisson counting statistics and definition of background also dominate data interpretation, and pose challenges to report values calibrated in biologically meaningful units (e.g., molecules, antibodies, size). First, an inspection of the fluorescence intensity distribution of many detected single molecules generally shows a Log-normal distribution^[25]^, which demonstrates that the photons detected to form that distribution are produced and detected stochastically, and that there are additional variabilities imposed by the measurement process. This means that intensity distributions of particles bearing one, two, three, or more fluorescent molecules can have significant overlap. Second, often the very sensitive detectors used for single-molecule detection have a limited dynamic range (the difference between the upper and lower LoD), and efforts to measure real biological samples, in which an individual EV may bear from zero to several hundred antibody molecules, can confront a limitation to measure brighter particles. Finally, while mathematical models can deconvolve such distributions post hoc to estimate the numbers of particles bearing zero, one, and two molecules (for example) or account for the effect of detector saturation for brighter particles, the ability to measure, rather than just detect, the estimated number of single molecules on a particle is subject to a number of assumptions and uncertainties.

From this perspective, the convergence of conventional flow cytometry technologies and the “top-down” measurement approach with high sensitivity, single molecule-based detection and the “bottom-up” approaches to measuring the low numbers of cargo molecules on many individual EVs is an exciting challenge in bioanalytical chemistry. As instrument sensitivities improve to strive for single molecule sensitivity, new challenges in high-throughput purification methods may need to be considered in order to remove all unbound label. With existing technologies, titration and dilution are usually sufficient to achieve this due to insensitivity to single molecules. Moreover, as other technologies are applied to measure single EVs, they will be challenged by many of these same issues, including (1) the development of appropriate concentration, size, and molecular cargo standards for single EV measurements; (2) the use of such standards to calibrate instrument response in absolute, rather than relative units, (3) the development of assays that report EV number, size, and molecular cargo in absolute units that can be compared across instruments and between labs; and (4) the sharing of instrument and assay methods and data in a manner that supports replication. These challenges must be solved to develop methods for EV measurement with the analytical rigor needed to understand their roles in health and disease, and to translate that understanding into useful new clinical diagnostics and therapeutic approaches.
